# Expression of aqueous cytokines and their correlation with retinal morphological biomarkers in nAMD patients

**DOI:** 10.1371/journal.pone.0342259

**Published:** 2026-02-11

**Authors:** Jingxin Zeng, Wenwen Shang, Pengfei Ren, Xiao Ke, Yonghao Zhao, Guina Liu, Jie Liu, Fang Lu, Xiaoshuang Jiang

**Affiliations:** 1 Department of Ophthalmology, West China Hospital, Sichuan University, Chengdu, China; 2 Chengdu Kanghong Pharmaceutical Group Company Limited, Chengdu, China; 3 Therapeutic Proteins Key Laboratory of Sichuan Provincial, Chengdu, China; Boston University School of Medicine, UNITED STATES OF AMERICA

## Abstract

**Objective:**

Neovascular age-related macular degeneration (nAMD) is characterized by pathological neovascularization in the macula, leading to irreversible central vision loss. This study aimed to evaluate cytokine profiles in the aqueous humor of nAMD patients and explore correlation between specific cytokines, clinical characteristics, and retinal morphological features.

**Methods:**

Our protocol was registered on Chinese Clinical Trial Registry (ChiCTR) under registration number ChiCTR2500106190 and approved by the Biomedical Research Ethics Committee of West China Hospital, Sichuan University (ID: 20231375). A total of 94 patients with nAMD and 34 cataract patients as the control group were enrolled. Aqueous humor samples were collected before anti-VEGF treatment or cataract surgery, and analyzed using cytometric bead array (CBA) for cytokines (MCP-1, ICAM-1, VCAM-1, CA-1, β-FGF, PPK/PK, and VEGF). Retinal structural biomarkers were assessed using optical coherence tomography (OCT), with statistical analysis performed to evaluate correlations between cytokine levels and retinal morphology.

**Results:**

Compared to cataract patients, nAMD patients exhibited elevated levels of CA-1, PPK/PK, ICAM-1, VCAM-1, and MCP-1 (P < 0.05). Baseline best-corrected visual acuity (BCVA) was reduced in nAMD patients with intraretinal fluid (IRF) or pigmentation compared to those without (P < 0.05). Visit BCVA also deteriorated in patients with fibrosis (P < 0.05). Additionally, central foveal thickness (CTR) was significantly thinner in patients with PED or SRF (P < 0.05). Visit BCVA demonstrated positive correlations with baseline BCVA (r = 0.689, P < 0.001). In contrast, the presence of fibrosis and elevated MCP-1 concentration was associated with a poorer visual outcome. In correlation analysis, β-FGF exhibited a negative correlation with CA-1, PPK, ICAM-1. VCAM-1 and MCP-1.

**Conclusion:**

In this cross-sectional study, the presence of IRF was significantly associated with reduced BCVA in nAMD patients. Baseline BCVA emerged as a critical predictor of visual prognosis in nAMD. Notably, retinal fibrosis development and elevated MCP-1 level linked to worse clinical outcomes, underscoring their potential as therapeutic targets. While recent anti-VEGF injections decreased VEGF levels in aqueous humor of nAMD patients, the impact of these agents on other cytokines remain unclear, suggesting a need for adjunct therapies to address multifaceted pathogenic pathways.

## Introduction

Neovascular age-related macular degeneration (nAMD) is the leading cause of irreversible central vision loss in individuals over 50 years old. By 2040, it is projected to affect over 170 million people worldwide, with more than half of the cases occurring in Asia. A recent consensus document classifies nAMD into Type 1, Type 2 and Type 3 neovascularization (NV) based on the severity of clinical manifestation observed in OCT scans [1]. Intravitreal anti-VEGF injection is the first-line treatment; however, despite therapy, the visual prognosis for most patients remains poor [2,3]. Approximately 59% of nAMD patients experienced disease recurrence within 1 year after discontinuing anti-VEGF treatment, while 40–70% of treated eyes developed macular fibrosis within 10 years [3].

Cytokine levels in the aqueous humor partially reflect those in the vitreous humor, making aqueous humor measurement a potential indicator of the activation of nAMD. Previous studies have shown significant association between nAMD and the concentration of monocyte chemoattractant protein-1 (MCP-1), intercellular adhesion molecule-1 (ICAM-1) and vascular cell adhesion molecule-1 (VCAM-1), showing that these inflammatory factors exhibited variable responses to anti-VEGF therapy [4–6]. This study aimed to evaluate the expression of seven cytokines associated with nAMD—CA-1, PPK/PK, ICAM-1, VCAM-1, basic fibroblast growth factor, MCP-1, and VEGF—in nAMD patients and age-related cataract patients as control. Additionally, optical coherence tomography (OCT) is a key tool for assessing nAMD severity, providing detailed visualization of both typical and atypical features of neovascularization at the RPE and subretinal level. These features include subretinal fluid (SRF), intraretinal fluid (IRF), pigment epithelial detachment (PED), subretinal hyperreflective material (SHRM), retinal fibrosis, and retinal pigmentation [7,8]. In this study, OCT was also used to analyze the correlation between these cytokines and retinal morphological characteristics to better understand their role in the pathogenesis of nAMD.

## Method

### Study subjects

The present research involved human participants and has been approved by the local ethical committee—West China Hospital of Sichuan University Biomedical Research Ethics Committee—and has been conducted according to the principles expressed in the Declaration of Helsinki. Written informed consent was obtained from the participants. Our Study did not include minors. This study included nAMD patients treated with anti-VEGF agents (ranibizumab, aflibercept, or conbercept) between October 1st, 2023, and October 1st, 2024, along with 34 control patients who underwent cataract surgery without any other ocular diseases. The study protocol was approved by the Institutional Review Board of West China Hospital. Comprehensive ophthalmic examinations were conducted prior to intravitreal injections, which included best-corrected visual acuity (BCVA) tests, slit-lamp examinations, fundus examinations, and intraocular pressure (IOP) measurements via noncontact tonometry. BCVA was assessed using a Snellen chart and converted to the logarithm of the minimum angle of resolution (logMAR) for analysis. Retinal morphological features including SRF, IRF, PED, SHRM, retinal fibrosis and retinal pigmentation were evaluated using optical coherence tomography (OCT) (Zeiss Cirrus HD-OCT 6000, CA, USA). Subretinal fluid (SRF) was characterized as a hyporeflective space situated between the retina and the retinal pigment epithelium (RPE). Hyperreflective foci (HRF) were defined as dot-like hyperreflective structures in neuroretina. Intraretinal fluid (IRF) refered to the abnormal accumulation of fluid within the layers of the neurosensory retina. Pigment epithelial detachment (PED) was characterized by separation and elevation of the retinal pigment epithelium from Bruch’s membrane. Subretinal hyperreflective material (SHRM) located between the neurosensory retina and the retinal pigment epithelium. Retinal fibrosis was characterized by formation of dysfunctional scar tissue between or under retina. Retinal pigmentation was characterized by the abnormal dispersion and aggregation of melanin granules from the retinal pigment epithelium.

Exclusion criteria included history of prior vitrectomy, rhegmatogenous retinal detachment, retinal vascular occlusion, uveitis, advanced glaucoma, complicated anterior segment surgery, lens dislocation, diabetic retinopathy and trauma.

### Sample collection

Aqueous humor samples were collected prior to every nAMD patient’s intravitreal injection or every cataract patient’s surgical procedure. Aqueous humor samples of nAMD patients were collected at least 1 month after anti-VEGF agent intravitreal injection. Approximately 0.1mL of aqueous humor was obtained via anterior chamber limbal paracentesis using a 30-gauge needle attached to an insulin syringe. Samples were immediately transferred to sterile plastic tubes and stored at −80°C until further analysis.

### Measurement of cytokines

Cytokine concentrations, including MCP-1, ICAM-1, VCAM-1, β-FGF and VEGF were determined using a cytometric bead array (CBA) (BD, CA, USA) according to the manufacturer’s instructions. CA-1 and PPK/PK were isolated and measured using protocols developed by the authors. Data acquisition was performed with a BD FACSCelesta™ Flow Cytometer (BD, CA, USA), and results were analyzed using FCAP Array™ Software Version 3.0 (BD, CA, USA). Standard curves for each cytokine were generated using reference concentrations provided in the kit, and cytokine concentrations were calculated accordingly.

### Retinal morphometric parameter analysis

logMAR BCVA and OCT biomarkers from nAMD patients were gathered during their initial hospital visit to establish baseline characteristics. Data acquisition was conducted independently by two ophthalmology residents who were blinded to each other’s assessments. In cases of disagreement between the two initial evaluators, a third senior ophthalmologist was consulted to provide a definitive evaluation. Data collected immediately before aqueous humor extraction were defined as current characteristics. Correlations and differences between these biomarkers, clinical characteristics, and the seven cytokines were analyzed.

### Statistical analysis

Statistical analyses were performed using SPSS version 21.0 software. Continuous data are presented as means ± standard deviations. The Shapiro–Wilk test was used to assess data normality. For normally distributed datasets, comparisons between groups were conducted using the t-test; for non-normally distributed data, the Mann–Whitney test was applied. Pearson correlation analysis was used for normally distributed data, and Spearman correlation analysis was employed for non-normally distributed data. A p-value of <0.05 was considered statistically significant.

## Results

### Baseline data

The baseline characteristics of the studied participants are summarized in Table 1. The nAMD group consisted of 94 patients with a mean age of 73.02 ± 10.88. The control group included 34 patients with a mean age of 63.74 ± 10.56. The age difference between the groups was statistically significant (P < 0.001). The male-to-female ratio was 1.69:1 in the nAMD group and 0.7:1 in the control group, while the OD/OS ratio was 1.34:1 and 1.13:1, respectively. The baseline logMAR BCVA was 0.90 ± 0.71 in the nAMD group and 0.83 ± 0.44 in the control group. No statistically significant differences were observed between the groups regarding sex distribution, eye laterality or baseline logMAR BCVA. (P > 0.05). Twelve in control group and 39 in nAMD group had a previous history of hypertension and were taking anti-hypertensive medication (P > 0.05). Six in control group and 12 in nAMD group had a previous history of diabetes melitus and were taking anti-diabetic medications (P > 0.05).

**Table 1:** Baseline Demographics of nAMD Group and Control Group.

Group	Total	Age (year)	Gender (M/F)	Eye (OD/OS)	BCVA (logMAR)	HBP(n)	DM(n)
control	34	63.74 ± 10.56	0.7:1	1.13:1	0.83 ± 0.44	12	6
nAMD	94	73.02 ± 10.88	1.69:1	1.34:1	0.90 ± 0.71	39	12
P	—	<0.001	0.029	0.827	0.866	0.47	0.51

P < 0.05 was considered statistically significant. HBP, high blood pressure. DM, diabetes mellitus.

### Measurement of aqueous humor cytokine level

The analysis indicated that, in comparison to the control group, CA-1, PPK/PK, ICAM-1, VCAM-1 and MCP-1 were significantly overexpressed in the nAMD group, whereas VEGF was significantly underexpressed (P < 0.05). There was no statistically significant difference in the expression levels of β-FGF between the two groups (P > 0.05). Detailed data can be found in Table 2.

**Table 2:** Comparison of Aqueous Cytokine Levels Between nAMD Group and Control group.

cytokines	control (pg/ml)	nAMD (pg/ml)	P
CA-1	2732.78 ± 9920.70	5357.72 ± 21426.12	0.009
PPK/PK	2527.48 ± 2483.27	4025.76 ± 3807.11	0.005
ICAM-1	48.45 ± 74.05	74.39 ± 98.95	0.044
β-FGF	15.58 ± 31.18	30.84 ± 67.38	0.649
VCAM-1	3448.55 ± 3227.05	7260.23 ± 9624.91	<0.001
VEGF	56.72 ± 95.14	17.46 ± 38.87	<0.001
MCP-1	52.90 ± 17.13	131.37 ± 374.89	0.027

### Retinal morphometric parameter analysis

At baseline, logMAR BCVA for patients with nAMD was 0.85 ± 0.69, and the average CRT was 278.91 ± 100.65µm. Among these patients, 45 exhibited SRF, as indicated by OCT images, and 49 showed IRF. Patients with IRF had significantly poorer BCVA compared to those without IRF (P < 0.05). Following anti-VEGF treatment, the average logMAR BCVA changed to 0.88 ± 0.56, and the average CRT decreased to 249.17 ± 80.96µm, with significant SRF and IRF improvement. Additional retinal biomarkers detected by OCT, such as PED, SHRM, fibrosis and retinal pigmentation, were also measured, their relationships with BCVA and CRT are detailed in Table 3.

**Table 3:** Analysis of Differences in Clinical Characteristics of nAMD Patients across Different Retinal Morphological Parameters.

Variable	n	Baseline Characteristics				n	Current characteristics			
		BCVA (logMAR)	*P*	CRT(μm)	*P*		BCVA (logMAR)	*P*	CRT(μm)	*P*
Overall	94	0.85 ± 0.69		278.91 ± 100.65		94	0.88 ± 0.64		249.17 ± 80.96	
IRF										
No		0.56 ± 0.52	0	250.47 ± 81.20	0.063		0.79 ± 0.56	0.106	242.65 ± 70.90	0.584
Yes	49	1.15 ± 0.73		296.24 ± 105.21		30	1.07 ± 0.77		262.44 ± 98.53	
PED										
No		0.96 ± 0.71	0.181	266.78 ± 81.24	0.526		0.89 ± 0.63	0.581	237.38 ± 78.04	0.025
Yes	35	0.76 ± 0.68		287.56 ± 116.60		28	0.86 ± 0.68		273.19 ± 82.92	
SRF										
No		0.99 ± 0.74	0.167	254.68 ± 77.41	0.083		0.82 ± 0.54	0.68	226.88 ± 72.08	0.005
Yes	45	0.79 ± 0.66		295.40 ± 110.44		34	0.98 ± 0.79		287.70 ± 82.07	
SHRM										
No		0.93 ± 0.76	0.866	263.35 ± 105.68	0.084		0.83 ± 0.61	0.157	236.98 ± 78.03	0.269
Yes	26	0.83 ± 0.53		298.96 ± 77.11		33	1.09 ± 0.79		267.27 ± 83.02	
Fibrosis		±								
No		0.81 ± 0.67	0.13	263.17 ± 90.07	0.631		0.68 ± 0.57	0	257.45 ± 71.57	0.518
Yes	38	1.05 ± 0.76		291.91 ± 105.18		42	1.15 ± 0.68		239.58 ± 90.66	
Pigmentation										
No		0.75 ± 0.58	0	274.75 ± 103.11	0.79		0.85 ± 0.60	0.355	255.83 ± 92.28	0.212
Yes	13	1.70 ± 0.81		284.08 ± 65.99		32	1.01 ± 0.75		237.63 ± 55.79	

IRF, intraretinal fluid; PED, pigment epithelial detachment; SRF, subretinal fluid; SHRM, subretinal hyperreflective material. CRT, central retinal thickness.

### Correlation analysis

After anti-VEGF treatment, logMAR BCVA was positively correlated with baseline logMAR BCVA (r = 0.689, P < 0.001), retinal fibrosis (r = 0.354, P = 0.001), and the cytokine MCP-1 level (r = 0.224, P = 0.033). As shown in Table 4.

**Table 4:** Analysis of Factors Related to Current BCVA (logMAR) in nAMD Patients.

Variable	current logMAR	
	r	P
Age (years)	0.165	0.115
Baseline logMAR	0.689	<0.001
Sex (M/F)	−0.03	0.776
IRF (No/Yes)	0.196	0.068
PED (No/Yes)	−0.036	0.737
SRF (No/Yes)	0.028	0.796
SHRM (No/Yes)	0.186	0.09
Fibrosis (No/Yes)	0.354	0.001
Pigmengtation (No/Yes)	0.002	0.987
CRT (μm)	0.03	0.782
CA-1 (pg/mL)	0.192	0.069
PPK/PK (pg/mL)	0.138	0.195
ICAM-1 (pg/mL)	0.175	0.094
β-FGF (pg/mL)	0.054	0.612
VCAM-1 (pg/mL)	0.124	0.245
VEGF (pg/mL)	−0.05	0.64
MCP-1 (pg/mL)	0.224	0.033

There were varying degrees of correlation among the aqueous humor cytokines. Notably, β-FGF exhibited a negative correlation with other cytokines. As shown in Table 5.

**Table 5:** Correlation analysis of aqueous cytokines in patients with nAMD.

	CA-1	PPK/PK	ICAM-1	β-FGF	VCAM-1	VEGF	MCP-1
	**r**	**r**	**r**	**r**	**r**	**r**	**r**
	**P**	**P**	**P**	**P**	**P**	**P**	**P**
CA-1	1	0.706**	0.713**	−0.352**	0.700**	0.401	0.725**
	–	0	0	0.001	0	0	0
PPK/PK		1	0.839**	−0.329**	0.754**	0.364**	0.714
		–	0	0.001	0	0	0
ICAM-1			1	−0.276**	0.789**	0.224**	0.737**
			–	0.008	0	0.032	0
β-FGF				1	−0.279**	−0.270**	−0.207**
				–	0.008	0.01	0.049
VCAM-1					1	0.198	0.786**
					–	0.061	0
VEGF						1	0.216**
						–	0.038
MCP-1							1
							–

**P < 0.05 was considered statistically significant.

Moreover, correlation analysis further indicated that baseline SHRM was weakly negatively associated with PPK, ICAM, and VCAM, yet positively associated with β-FGF. Weak positive associations were also noted for baseline pigmentation with CA1 and VEGF, and for visit pigmentation with PPK and VCAM. Conversely, neither baseline nor visit CRT demonstrated any statistically significant associations with the cytokine profiles analyzed (Supplement 1).

## Discussion

Neovascular age-related macular degeneration (nAMD) is a leading cause of severe, irreversible central vision loss in the elderly population globally, and is characterized by the pathological growth of new, leaky blood vessels from the choroid into the subretinal space, a process driven by an upregulation of vascular endothelial growth factor (VEGF). This choroidal neovascularization leads to exudation, hemorrhage, and ultimately, fibrotic scarring. The mean age of the nAMD patients was greater than that of controls because the indications for anti-VEGF injection in nAMDs predominantly affect older patients. Besides, to date, it remains unclear whether the expression profile of cytokines in the aqueous humor changes with age [9].

During ocular inflammation, lymphocytes and macrophages are recruited to the retinal pigment epithelium, where they release proinflammatory cytokines that significantly contribute to nAMD pathogenesis [10,11]. For instance, MCP-1 facilitates leukocyte recruitment to sites of injury during angiogenesis [12,13]. ICAM-1 and VCAM-1 are mainly expressed by endothelial cells (though ICAM-1 is also expressed at a low level by immune cells), and are critical for leukocyte transport during inflammation [14,15]. In our study, the concentrations of ICAM-1, VCAM-1, and MCP-1 in the aqueous humor of nAMD patients were significantly higher compared to those in control group, suggesting that leukocyte migration influences the activity of new blood vessels in the macular area. These findings imply that inhibiting ICAM-1, VCAM-1, and MCP-1 could help protect the retina from inflammatory damage. Correlation analysis further revealed that MCP-1 concentration was positively correlated with visual acuity following anti-VEGF treatment, suggesting that MCP-1 may serve as a predictor of poor visual prognosis in nAMD patients. Moreover, we found that ICAM-1 and VCAM was weakly negatively associated with baseline SHRM, which located between the neurosensory retina and the retinal pigment epithelium, but not correlated with visit SHRM.

CA-1 is a common metalloprotein found in high concentration in red blood cells [16]. PPK/PK (plasma prekallikrein/plasma kallikrein) acts as a key regulator of cardiovascular dynamics, complement activation, and inflammatory responses. In this study, the concentrations of CA-1 and PPK/PK in the aqueous humor of nAMD patients were significantly higher than those in the control group. Correlation analysis revealed a positive correlation between CA-1 and PPK/PK (r = 0.706, P < 0.001). Moreover, we found that CA-1 was positively associated with baseline pigmentation. This suggests that CA-1 may be associated with vascular rupture and hemorrhage in nAMD, which is a finding consistent with reports on elevated extracellular CA-1 levels in hemorrhagic retinal injuries [17,18]. The results indicate that targeting downstream proteins of CA-1 may help alleviate vascular permeability.

β-FGF, expressed in nearly all retinal cells, plays significant roles in protecting photoreceptors, enhancing RPE phagocytic activity, inducing retinal regeneration, and promoting angiogenesis and fibrosis [19–21]. In our study, β-FGF concentration in aqueous humor was not significantly different between nAMD and control group. Correlation analysis revealed that β-FGF was negatively correlated with all other cytokines and baseline SHRM. Its low concentration in the nAMD group may reflect the restricted area of macular involvement.

In our study, VEGF concentration was lower in nAMD patients than that in the control group, possibly due to the suppressive effect of anti-VEGF agents, highlighting the significance of VEGF in treating nAMD. This finding aligns with previous reports by Chan WM and Jonas JB [22,23]. Although aqueous humor samples were collected a minimum interval of one month after last anti-VEGF treatment. VEGF concentration remained low in aqueous samples, indicating that anti-VEGF agents have a prolonged pharmacologic activity [24].

Patients with IRF at baseline have poorer vision and a thicker retinal central layer, which is positively correlated with logMAR BCVA after anti-VEGF treatment. This suggests that IRF at baseline is associated with poor visual quality and is a key factor in the poor treatment outcomes. IRF not only correlates with baseline vision loss but also increases the risk of fibrosis, making it a significant prognostic biomarker [25,26].

Subretinal fibrosis is considered the strongest predictor of final visual acuity. Its pathogenesis involves chronic inflammation, angiogenesis and abnormal wound healing processes, with contributing factors such as disease chronicity, aging, smoking, oxidative stress, prolonged anti-VEGF therapy and genetic predisposition [23]. For instance, aging enhances subretinal fibrosis by activating and mobilizing circulating fibrocytes and bone marrow-derived macrophages to infiltrate the lesion site in the fundus of older adults [9]. Macrophages promote subretinal fibrosis through the production of TGFβ [27]. Extensive literature supports the critical role of epithelial-mesenchymal transformation (EMT) of RPE in myofibroblast generation in subretinal fibrosis of nAMD, and the reciprocal activation between Wnt5a and β-catenin mediates EMT as a key driver of subretinal fibrosis in nAMD [28].

Yu E et al. identified that apatinib, a highly selective inhibitor of VEGFR2 tyrosine kinase, has the capability to suppress nAMD angiogenesis and fibrosis by downregulating STAT3 phosphorylation [29]. Zhang Q found that RGS1 is closely associated with the proliferation of vascular endothelial cells, making both potential new targets for future AMD treatment [30].

Our study has several limitations. The study was not prospective. We collected aqueous humor sample from nAMD patients at least 1 month after last anti-VEGF treatment, but we didn’t re-collect their aqueous humor to study the change of cytokines. We correlated MCP-1 level and retinal fibrosis with poor visual acuity in nAMD patients, but the small sample size may have prevented us from obtaining significant results. A future prospective study with a large number of either anti-VEGF treated and untreated patients is necessary to determine the association between MCP-1 concentration, retinal fibrosis and retinal imaging biomarkers in nAMD patients.

## Conclusion

This study demonstrated that aqueous humor from nAMD patients exhibited elevated levels of CA-1, PPK/PK, ICAM-1, VCAM-1, MCP-1 and β-FGF compared to the control group, supporting the inflammation-driven pathogenesis of nAMD. IRF at baseline was associated with poor visual quality, while post-treatment outcomes were influenced by baseline best corrected visual acuity, retinal fibrosis, and MCP-1 levels. Retinal fibrosis and MCP-1 may therefore serve as potential therapeutic targets in managing nAMD.

## Supporting information

S1 Table Correlation analysis of OCT markers and cytokines in nAMD patients. (DOCX)
