# Supplementary material for: Expression of aqueous cytokines and their correlation with retinal morphological biomarkers in nAMD patients
Source: PLoS One. 2026 Feb 11;21(2):e0342259. doi: 10.1371/journal.pone.0342259 (PMC12893568; doi:10.1371/journal.pone.0342259)
Supplement: S1 Table — (DOCX) [file pone.0342259.s001.docx]

Supplemet 1 Correlation analysis of OCT markers and cytokines in nAMD patients.

|  | CA1 | PPK | ICAM | bFGF | VCAM | VEGF | MCP1 |
| --- | --- | --- | --- | --- | --- | --- | --- |
|  | r | r | r | r | r | r | r |
|  | P | P | P | P | P | P | P |
| baseSRF | -0.132 | 0.042 | -0.013 | 0.053 | -0.034 | -0.06 | -0.01 |
|  | 0.226 | 0.703 | 0.902 | 0.631 | 0.754 | 0.583 | 0.927 |
| basePED | 0.133 | 0.011 | -0.058 | 0.113 | -0.134 | -0.094 | -0.131 |
|  | 0.223 | 0.92 | 0.595 | 0.302 | 0.219 | 0.389 | 0.225 |
| baseIRF | 0.097 | 0.152 | 0.033 | 0.057 | -0.034 | 0.059 | 0.005 |
|  | 0.373 | 0.161 | 0.762 | 0.605 | 0.757 | 0.585 | 0.961 |
| baseSHRM | -0.094 | -.251** | -.237** | .275** | -.238** | -0.126 | -0.107 |
|  | 0.397 | 0.022 | 0.031 | 0.012 | 0.03 | 0.254 | 0.331 |
| baseFibrosis | 0.074 | 0.053 | -0.052 | .218** | 0.025 | -0.064 | -0.14 |
|  | 0.506 | 0.635 | 0.638 | 0.047 | 0.818 | 0.56 | 0.202 |
| basePigment | .310** | -0.119 | -0.092 | 0.081 | -0.117 | .266** | 0.169 |
|  | 0.004 | 0.277 | 0.402 | 0.46 | 0.285 | 0.013 | 0.12 |
| visitSRF | -0.089 | 0.129 | 0.051 | -0.061 | -0.006 | 0.019 | -0.118 |
|  | 0.417 | 0.236 | 0.64 | 0.574 | 0.959 | 0.863 | 0.277 |
| visitPED | 0.16 | -0.056 | -0.099 | -0.03 | -0.194 | 0 | -0.114 |
|  | 0.142 | 0.612 | 0.365 | 0.782 | 0.074 | 0.998 | 0.295 |
| visitIRF | -0.128 | 0.116 | 0.01 | -0.03 | 0.009 | 0.013 | -0.097 |
|  | 0.242 | 0.287 | 0.927 | 0.785 | 0.933 | 0.903 | 0.371 |
| visitSHRM | 0.175 | -0.09 | -0.092 | 0.05 | 0.025 | 0.064 | -0.106 |
|  | 0.107 | 0.408 | 0.398 | 0.65 | 0.816 | 0.553 | 0.329 |
| visitFibrosis | -0.154 | -0.076 | -0.193 | 0.102 | -0.071 | -0.135 | -0.154 |
|  | 0.157 | 0.486 | 0.075 | 0.348 | 0.515 | 0.211 | 0.155 |
| visitPigment | -0.038 | .214** | 0.165 | -0.153 | .320** | -0.046 | -0.085 |
|  | 0.726 | 0.048 | 0.128 | 0.16 | 0.003 | 0.672 | 0.431 |
| baseCRT | 0.176 | 0.11 | 0.089 | 0.111 | 0.023 | 0.099 | 0.022 |
|  | 0.12 | 0.334 | 0.437 | 0.331 | 0.839 | 0.384 | 0.845 |
| visitCRT | -0.106 | -0.063 | -0.186 | 0.04 | -0.202 | 0.124 | -0.188 |
|  | 0.337 | 0.57 | 0.089 | 0.719 | 0.065 | 0.26 | 0.085 |

**P<0.05 was considered statistically significant.
